# Transfer learning for honey bee toxicity prediction: MolFormer versus classical QSAR representations

**DOI:** 10.1007/s10646-026-03149-x

**Published:** 2026-09-01

**Authors:** Alan Victor de Souza Pinho, Edilson Beserra de Alencar Filho, Rosalvo Ferreira de Oliveira Neto

**Affiliations:** 1 Undergraduate Program in Computer Engineering, Federal University of San Francisco Valley, Antônio C. Magalhães Avenue - 510, Santo Antonio, Juazeiro, 48902-300 Bahia Brazil; 2 Graduate Program in Health and Biological Sciences, Graduate Program in Biosciences, Federal University of San Francisco Valley, José de Sá Maniçoba Avenue, Downtown, Petrolina, 56304-917 Pernambuco Brazil; 3 Undergraduate Program in Pharmaceutical Sciences, Federal University of San Francisco Valley, José de Sá Maniçoba Avenue, Downtown, Petrolina, 56304-917 Pernambuco Brazil

**Keywords:** Transfer learning, QSAR, Toxicity prediction, *Apis mellifera*

## Abstract

Honey bee (*Apis mellifera*) toxicity assessment is essential for environmental risk evaluation of agrochemicals, yet experimental data are costly and often limited, constraining the development of robust predictive models. This study investigates whether transfer learning via pretrained chemical language models can provide competitive QSAR performance for bee toxicity classification. Using the ApisTox dataset (1,035 compounds; 296 toxic and 739 non-toxic for bees), molecular representations were derived from SMILES in three ways: (i) PaDEL molecular descriptors, (ii) RDKit Morgan fingerprints, and (iii) MolFormer embeddings extracted from a publicly available reduced-scale pretrained checkpoint (~ 100 M molecules; ~10% ZINC + ~ 10% PubChem), used as frozen features. Three classical classifiers: Random Forest, Support Vector Machine, and Multilayer Perceptron were trained and evaluated under 5-fold cross-validation. Fingerprints paired with RF achieved the best overall discrimination, as measured by the Area Under the Receiver Operating Characteristic (ROC-AUC = 0.866). Importantly, MolFormer embeddings combined with SVM reached near-parity (ROC-AUC = 0.859) and consistently outperformed PaDEL descriptors for all classifiers (ΔROC-AUC = + 0.005 to + 0.021). These results demonstrate that transfer-learned chemical embeddings can rival established QSAR baselines while simplifying feature engineering, supporting their practical adoption for ecotoxicological screening under limited labeled data.

## Introduction

Quantitative Structure–Activity Relationship (QSAR) modeling is a well-established paradigm in computational toxicology, enabling the prediction of biological and toxicological properties of chemical compounds from their molecular structure. Over the past decades, QSAR approaches have been extensively applied in drug discovery, environmental safety, and regulatory science, providing cost-effective and ethically sound alternatives to experimental testing (Cherkasov et al. 2014; Tropsha 2010; Hung and Gini 2021). In environmental risk assessment, QSAR models support the prioritization and screening of chemicals under international regulatory frameworks, contributing to the reduction of animal testing and accelerating decision-making processes (OECD 2014).

A particularly critical application of QSAR lies in the assessment of agrochemical toxicity to non-target organisms. Among these, *Apis mellifera* (the Western honey bee) has received special attention due to its essential role in pollination, biodiversity preservation, and global food security. Despite their ecological and economic importance, honey bee populations have been declining worldwide, with pesticide exposure identified as a major contributing factor, together with habitat loss and climate change (Potts et al. 2010). This context highlights the urgent demand for reliable computational models capable of predicting bee toxicity and supporting the development and regulation of safer chemical products.

The urgency of developing reliable computational tools for pollinator toxicity assessment is further underscored by international regulatory frameworks. The European Food Safety Authority (EFSA) has issued guidance specifically addressing the risk assessment of plant protection products to bees (EFSA 2013), and the Organisation for Economic Co-operation and Development (OECD) provides standardized test guidelines for acute oral toxicity (OECD 1998a) and acute contact toxicity (OECD 1998b) in honeybees. These regulatory instruments highlight the critical role of both experimental and predictive toxicology in ensuring the safety of agrochemicals for non-target pollinator species. In silico approaches capable of reliably screening large chemical libraries for bee toxicity potential are therefore not only scientifically relevant but increasingly demanded by the regulatory landscape.

Transfer learning has emerged as an effective strategy to address a fundamental limitation of deep learning in cheminformatics: the scarcity of large, labeled experimental datasets. Rather than training models entirely from scratch on limited task-specific data, transfer learning enables the reuse of representations learned from large-scale pretraining corpora, improving model performance and generalization when only limited labeled data are available (Simões et al. 2018; Cai et al. 2020). This paradigm has achieved notable success in computer vision and natural language processing, and has more recently gained substantial visibility in computational chemistry and drug discovery, with applications spanning physicochemical property prediction (Yao et al. 2024), biological activity modeling (Li and Fourches 2020), molecular generation (Segler et al. 2018), and virtual screening (Zhang et al. 2024), reflecting the broader expansion of artificial intelligence across drug discovery pipelines (Zhong et al. 2018).

Formally, transfer learning operates through the alignment of source and target domains and tasks, where the domain D is defined by a feature space and a marginal probability distribution, and the task T is characterized by a label space and a predictive function (Pan and Yang 2010; Cai et al. 2020). In cheminformatics, source domains typically consist of large, diverse molecular datasets, while target domains correspond to specific biological or toxicological endpoints with limited labeled instances. Transfer strategies can be categorized as instance-based, parameter-based, relation-based, or feature-based. The latter, wherein a pretrained model is used as a fixed feature extractor to generate transferable molecular representations, is particularly well-suited to QSAR scenarios where labeled data are constrained, as it decouples representation learning from downstream supervised modeling (Cai et al. 2020; Simões et al. 2018). Chemical language models, pretrained on large SMILES corpora, have emerged as a powerful implementation of this feature-based paradigm, generating dense molecular embeddings that implicitly encode structural, semantic, and physicochemical information without requiring manual feature engineering.

Within this framework, the present study investigates whether transfer learning can be efficiently applied to QSAR modeling for predicting toxicity to *Apis mellifera*. Molecular embeddings extracted from pre-trained chemical language models were used as input features to classical machine learning algorithms and systematically compared with baseline models built using PaDEL molecular descriptors (Yap 2011) and RDKit (Landrum et al. 2025) fingerprints. Toxicity data were obtained from the ApisTox database, a recently released open-access and curated resource containing LD₅₀ values and molecular structures for hundreds of compounds relevant to honey bee toxicity (Adamczyk et al. 2025). By benchmarking learned embeddings against traditional representations, this study aims to clarify the practical value of transfer learning for ecotoxicological QSAR and to contribute to more robust computational tools for pollinator protection.

## Methods

### Dataset

Toxicity data for 1,035 agrochemicals and pesticides were collected from a spreadsheet available in the online ApisTox platform (Adamczyk et al. 2024), accessible at https://zenodo.org/records/13350981. The dataset comprises 296 compounds labeled as toxic and 739 labeled as non-toxic to honey bees (*Apis mellifera*), following the curation protocol described by the authors. For each compound, the dataset provides the corresponding SMILES representation along with a binary toxicity label indicating the absence (0) or presence (1) of toxicity and other indicative data.

The toxicity annotation is based on the methodology adopted by the United States Environmental Protection Agency (EPA), which defines toxicity thresholds using acute LD₅₀ values (Adamczyk et al. 2025; U.S. EPA, 2014). Specifically, a compound is classified as toxic if its LD₅₀ value is less than or equal to 11 µg/bee, considering either oral or contact exposure routes. Compounds with LD₅₀ values above this threshold are classified as non-toxic. This binary categorization reflects regulatory practice and provides a consistent criterion for computational modeling of bee toxicity.

It should be noted that the ApisTox dataset provides two complementary labeling schemes: a binary classification (toxic / non-toxic), defined by the LD₅₀ threshold described above, and a 3-level ternary classification system (Highly Toxic / Moderately Toxic / Non-Toxic), as detailed in the original dataset publication (Adamczyk et al. 2025). The present study employs exclusively the binary labeling scheme, which aligns with EPA regulatory practice and enables direct comparison with existing QSAR models for bee toxicity. Exploration of the ternary classification scheme represents a relevant direction for future work.

The ApisTox dataset integrates acute toxicity information from multiple curated and authoritative sources, including the ECOTOX database maintained by the United States Environmental Protection Agency (Olker et al. 2022), the Pesticide Properties DataBase (PPDB), and the Bio-Pesticides DataBase (BPDB) (Lewis et al. 2016). Oral, contact, and other exposure endpoints are considered, and for each compound the most toxic exposure route is retained (Adamczyk et al. 2025; U.S. EPA, 2014). This conservative strategy ensures that the dataset captures the highest potential risk posed by each chemical to pollinators.

By aggregating toxicity data from diverse experimental sources and harmonizing them under a unified labeling scheme, ApisTox constitutes a comprehensive benchmark for the assessment of small-molecule toxicity to honey bees. Its combination of curated molecular structures, standardized toxicity labels, and ecotoxicological relevance makes it particularly suitable for the development, evaluation, and comparison of QSAR models in environmental toxicology and pollinator risk assessment (Puzyn et al. 2010).

### MolFormer as feature extractor

The methodology adopted in this study is grounded in the feature-based transfer learning paradigm. Specifically, MolFormer (Ross et al. 2022), a large-scale chemical language model developed by IBM, was employed as a frozen feature extractor to derive molecular embeddings directly from SMILES representations. In this setting, the encoder layers of the pretrained model act as sophisticated transformation functions that map each SMILES string to a 768-dimensional dense vector, capturing complex syntactic and semantic properties of the corresponding molecular structure without any task-specific fine-tuning. These embeddings were subsequently used as fixed input features for the classical machine learning classifiers described in Sect.  2.4.

This approach eliminates the need for integrating multiple descriptor calculation libraries and bypasses explicit feature selection steps, substantially simplifying the modeling pipeline while preserving rich, structurally meaningful information learned from large-scale pretraining data.

### MolFormer

**Fig. 1:**
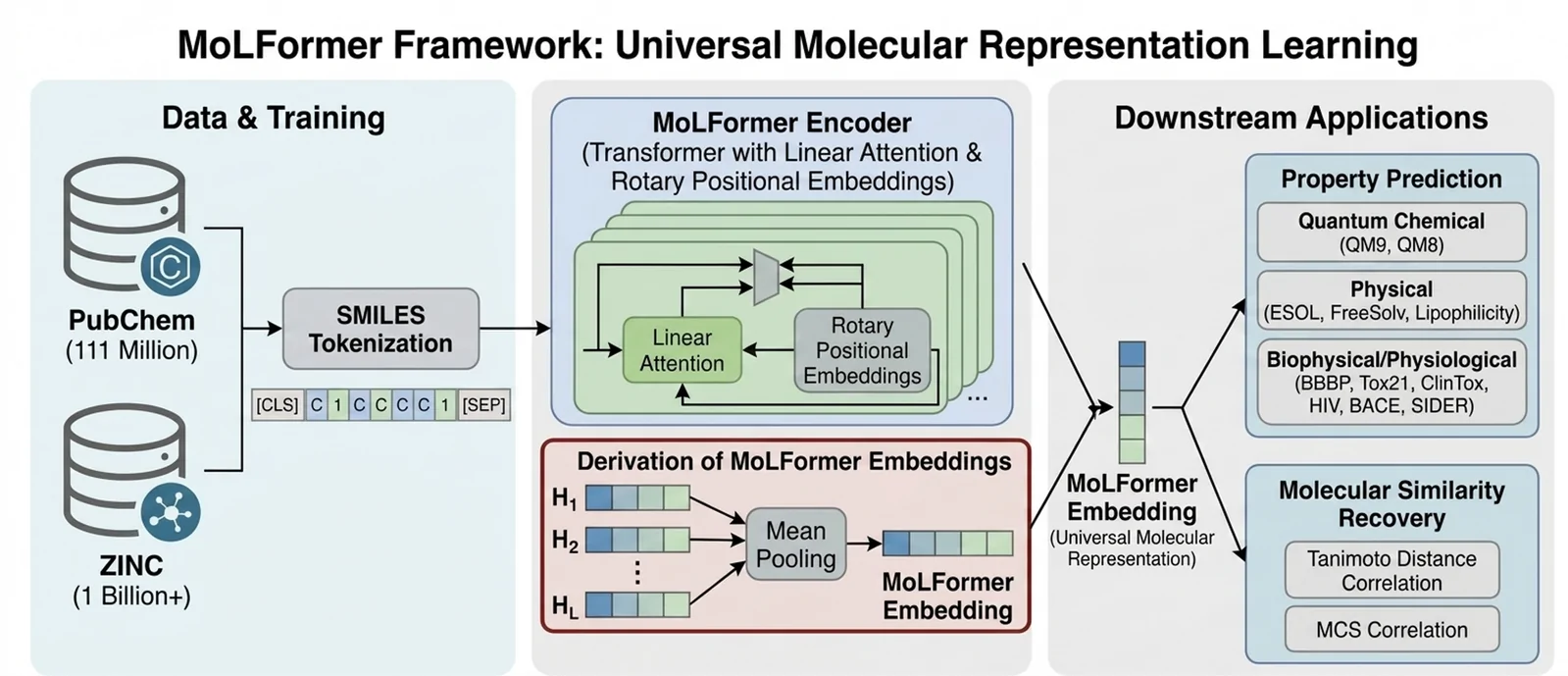
Overview of the MoLFormer framework. The model is trained using large-scale chemical data (SMILES) from the PubChem (111 million) and ZINC (1.1 billion+) databases. The SMILES sequences are tokenized and processed by the MoLFormer Encoder, which utilizes a linear attention mechanism coupled with rotary positional embeddings for efficiency and scalability

MolFormer is a large-scale chemical language model specifically designed to improve accuracy and robustness in molecular property prediction tasks. It is built upon the Bidirectional Encoder Representations from Transformers (BERT) architecture and adapts its core principles to the chemical domain by treating SMILES strings as a structured language (Ramos et al. 2025). The overall architecture and data flow of the MoLFormer framework are illustrated in Fig. 1. By leveraging self-attention mechanisms, MolFormer is able to model long-range dependencies and contextual relationships between atomic symbols, functional groups, and structural motifs encoded within SMILES representations.

The model is trained in a fully unsupervised manner using a masked language modeling objective, where tokens in SMILES sequences are randomly masked and the model is trained to predict the missing elements based on their surrounding context (Ross et al. 2022). This strategy enables MolFormer to learn general and transferable representations of chemical structures without relying on labeled property data. The most comprehensive variant, known as MolFormer-XL, was pre-trained on a massive corpus of over 1.1 billion unique molecules curated from the PubChem and ZINC databases, making it one of the largest pre-training efforts reported for chemical language models (Ross et al. 2022). This scale allows the model to capture a broad and diverse coverage of chemical space, encompassing a wide range of molecular sizes, functional groups, and structural patterns.

Extensive benchmarking has demonstrated that MolFormer achieves state-of-the-art performance across multiple molecular property prediction tasks. In particular, MolFormer-XL outperformed several graph neural network (GNN) architectures on both classification and regression benchmarks from the MoleculeNet suite, which includes datasets spanning physicochemical, biological, and toxicological endpoints (Ross et al. 2022). MolFormer has shown strong predictive capability for quantum mechanical properties, indicating that its learned embeddings implicitly encode complex spatial and electronic relationships between atoms, despite relying solely on linear SMILES input rather than explicit three-dimensional molecular graphs.

A key factor underlying MolFormer’s performance is the introduction of architectural optimizations tailored to molecular sequences. Among these, the use of rotary positional embeddings represents a significant advancement over conventional absolute positional encodings. Rotary embeddings encode positional information through relative rotations in embedding space, enabling the model to more effectively represent ordering and distance relationships between tokens (Ross et al. 2022). In the context of SMILES, this enhancement improves the model’s ability to capture ring closures, branching patterns, and long-range dependencies that are critical for accurate molecular representation.

In addition to the MolFormer-XL architecture described in the literature, the official implementation makes available pretrained checkpoints trained on a reduced-scale corpus of approximately 100 million molecules, corresponding to about 10% of the combined ZINC and PubChem datasets. These publicly released checkpoints provide a practical trade-off between computational cost and representational quality, enabling MolFormer-based embeddings to be used in downstream QSAR pipelines under limited hardware resources. At the time of writing, the full MolFormer-XL checkpoints trained on the complete 1.1B-molecule corpus are not publicly available.

Within the scope of QSAR modeling, MolFormer serves as a powerful feature extractor for transfer learning. The embeddings generated by its encoder layers provide dense, information-rich molecular representations that can be directly used as input to classical machine learning algorithms. This approach avoids the need for handcrafted descriptor calculation and explicit feature selection, while still retaining chemically meaningful information learned from large-scale data. Consequently, MolFormer offers a flexible and effective framework for incorporating transfer learning into predictive toxicology, particularly in data-limited scenarios such as honey bee toxicity assessment.

### Training models

**Fig. 2:**
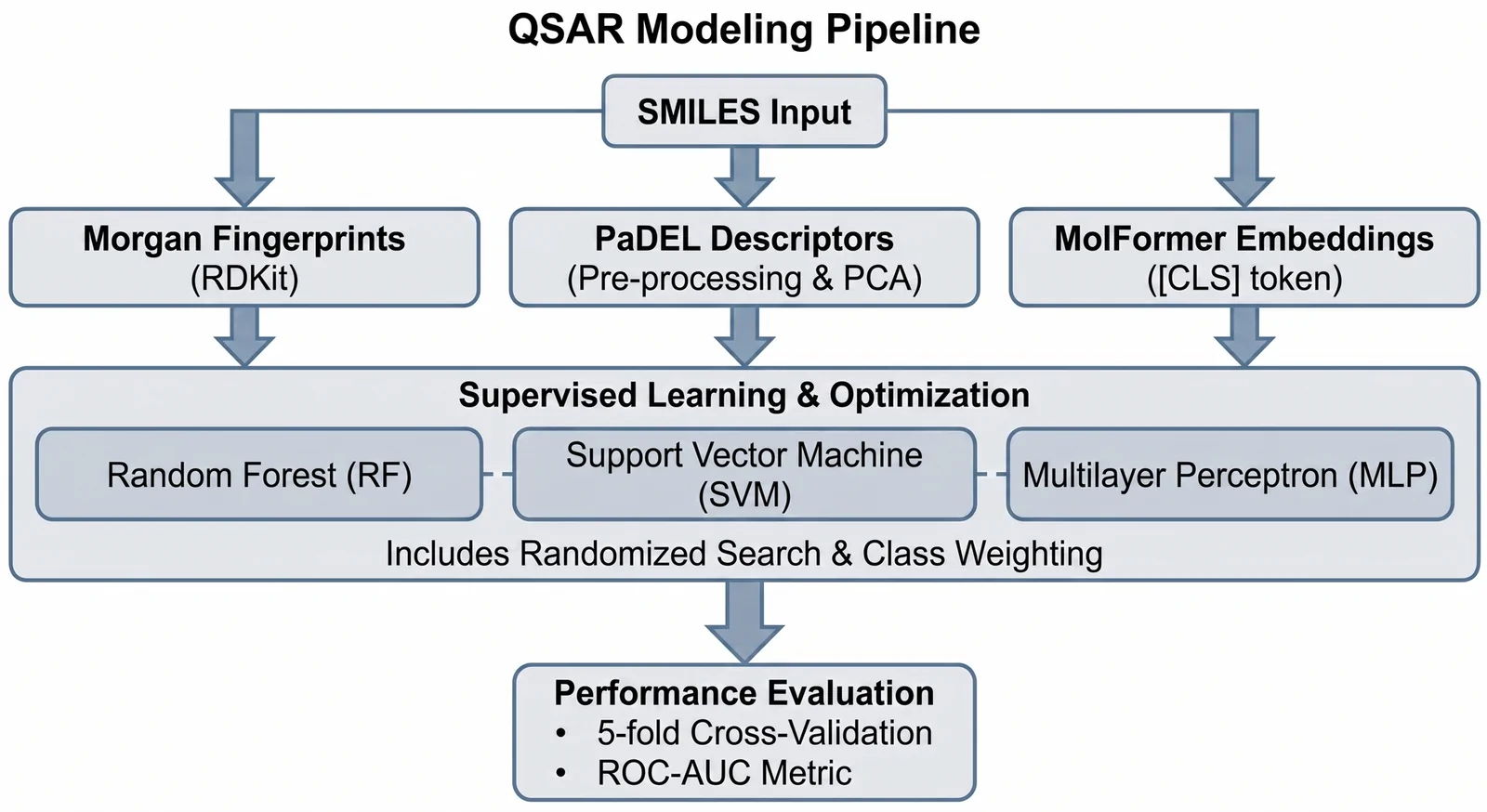
Workflow of the QSAR modeling pipeline. From SMILES, three molecular representations are generated (Morgan fingerprints, PaDEL descriptors and MoLFormer embeddings) and used to train RF, SVM and MLP models. Performance is assessed by 5-fold cross-validation using ROC-AUC

The integrated pipeline for feature extraction, model training, and evaluation adopted in this study is illustrated in Fig. 2. Three supervised learning algorithms were considered in this paper due to their established use in QSAR and robustness under high-dimensional representations: Random Forest (RF), Support Vector Machine (SVM), and Multilayer Perceptron (MLP). All models were trained using three alternative molecular representations derived from the same SMILES input:

1. Morgan fingerprints computed with RDKit,
2. PaDEL molecular descriptors (with preprocessing and PCA applied only to this branc
3. MolFormer embeddings were extracted from the last hidden layer (768 dimensions) using the publicly available reduced-scale pretrained checkpoint (~100M molecules), and used as fixed features.

Hyperparameter optimization was performed for RF and SVM using randomized search as implemented in scikit-learn (Pedregosa et al. 2011). For RF, the search explored the number of trees (200–1000), optional limitation of maximum depth (up to 50 levels), and regularization-related parameters controlling split criteria and node sizes (e.g., max_features and minimum samples per node). For SVM, the search targeted the RBF kernel parameters C (0.01–1000) and γ (log-scale and automatic settings), which govern margin penalization and decision boundary flexibility, respectively. In both cases, the Area Under the Receiver Operating Characteristic Curve (ROC-AUC) was used as the selection criterion for the best configuration.

To mitigate class imbalance (toxic vs. non-toxic), RF and SVM were trained with class weighting (class_weight = “balanced”). SVM models were fitted within a pipeline that included standardization (StandardScaler) to ensure comparable feature scales.

The MLP was trained using a fixed configuration (without randomized search): one dense hidden layer with 768 neurons and ReLU activation, followed by a single sigmoid output neuron for binary toxicity probability. Weights were initialized with Glorot uniform initialization and optimized using Adam (learning rate 3 × 10^− 4^) for 50 epochs with batch size 8. Class weights were also applied, and the best model weights were retained based on validation loss monitoring.

### Performance evaluation

Model performance was primarily assessed using the ROC-AUC, which quantifies class separability independently of a fixed decision threshold and is particularly appropriate for imbalanced datasets. This threshold-independent nature renders ROC-AUC especially suitable for comparative benchmarking across heterogeneous classifiers and molecular representations, where probability score distributions may differ substantially between approaches. ROC-AUC values were interpreted relative to the random baseline (0.5) and the ideal classifier (1.0) (Fawcett 2006).

Validation employed 5-fold cross-validation (Kohavi 1995). In each fold, models were trained on k − 1 partitions and evaluated on the held-out partition; final performance was summarized by averaging the metric across folds. Hyperparameter selection for RF and SVM followed the ROC-AUC maximization criterion. The exclusive use of cross-validation as the evaluation protocol, without a separate held-out test set, was a deliberate methodological choice motivated by the limited size of the minority class (296 toxic compounds). Reserving a fixed test partition would have substantially reduced training set size and increased variance in performance estimates. The 5-fold cross-validation scheme ensures that every compound serves as a held-out evaluation instance exactly once, providing comprehensive coverage of the dataset under conditions equivalent to repeated hold-out testing, consistent with established QSAR validation practice (Kohavi 1995; Tropsha 2010; Cherkasov et al. 2014).

## Results

**Fig. 3:**
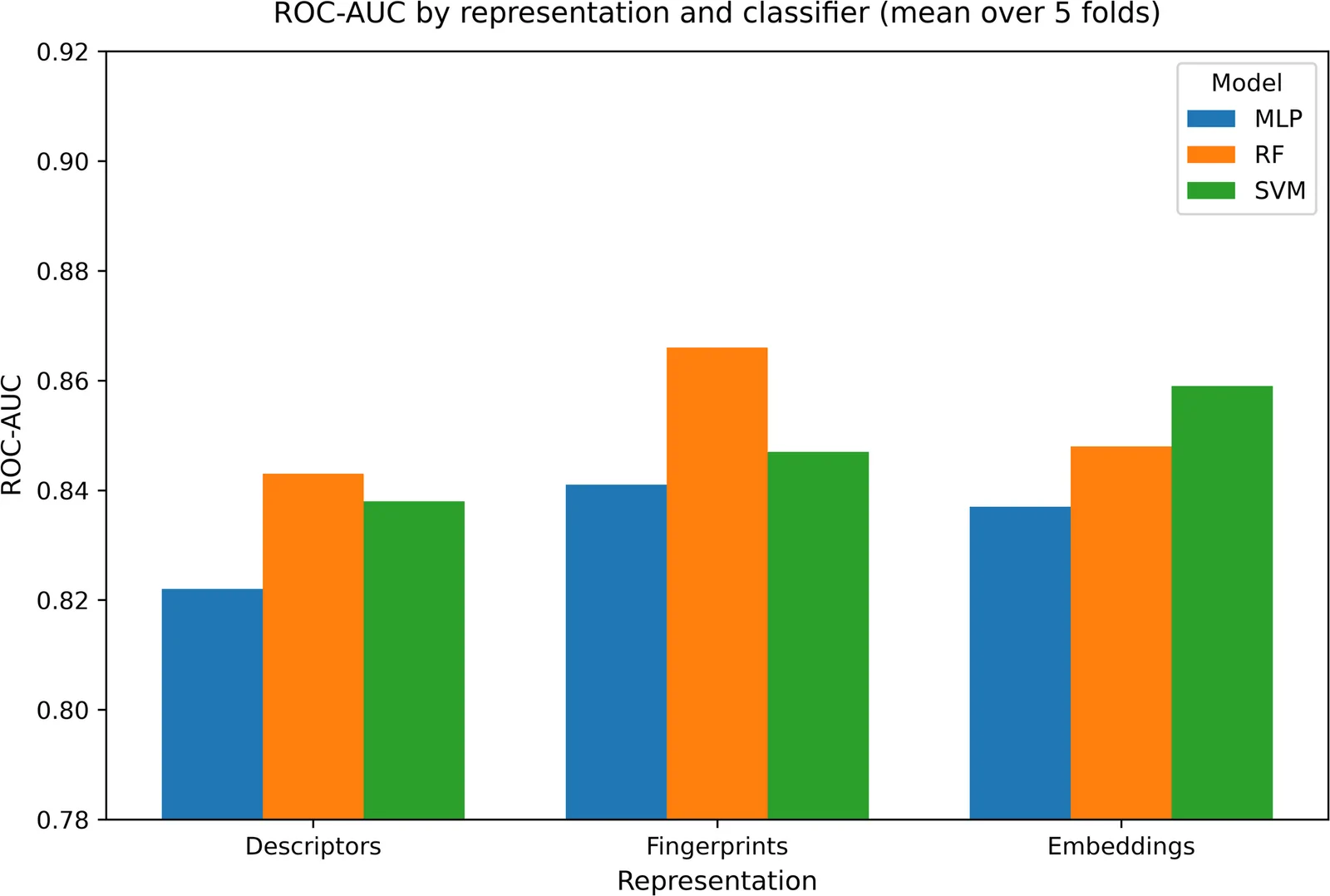
ROC-AUC by representation and classifier (mean over 5 folds)

Figure 3; Table 1 provide a compact overview of the mean ROC-AUC obtained by each representation–classifier pair. Overall performance reflects a clear interaction between representation and classifier: Morgan fingerprints remain a strong baseline, whereas MolFormer embeddings achieve highly competitive results with reduced resource complexity. The best mean ROC-AUC is obtained by Random Forest with Morgan fingerprints (ROC-AUC = 0.866), confirming the effectiveness of substructure-based representations for ApisTox. It is important to highlight that the combination of SVM with MolFormer embeddings achieves performance almost equivalent to the best overall baseline (RF-fingerprints) (ROC-AUC = 0.859, Δ = 0.007). This result is particularly noteworthy because the embeddings were extracted from a small-scale checkpoint of publicly available MolFormer, trained on only 10% of the ZINC and PubChem databases. In practical terms, a comparatively lightweight pretraining regime already yields a representation that can closely match classical QSAR pipelines on this ecotoxicological benchmark. This naturally motivates an important question: If embeddings derived from a full-scale checkpoint trained on the reported ~ 1.1B molecules were available, to what extent might the remaining performance gap be reduced, or potentially inverted? Although this is not evaluated in this work due to lack of access to the complete database, the current result already demonstrates a high potential for data transfer learning in predicting toxicity with limited data.

As shown in Table 2, MolFormer embeddings outperform PaDEL descriptors across all three classifiers, yielding consistent ROC-AUC gains (+ 0.005 to + 0.021). The largest improvement is observed for SVM (+ 0.021), suggesting that MolFormer’s learned representation is particularly well aligned with margin-based decision boundaries. Morgan fingerprints also outperform PaDEL across classifiers.

**Table 1:** ROC-AUC by representation and classifier (mean over 5 folds)

Representation	Random Forest	SVM	MLP
Descriptors (PaDEL)	0.843	0.838	0.822
Fingerprints (Morgan)	**0.866**	0.847	0.841
Embeddings (MolFormer)	0.848	**0.859**	0.837

**Table 2:** ΔROC-AUC relative to PaDEL descriptors (same classifier)

Classifier	Fingerprints − Descriptors	Embeddings − Descriptors
Random Forest	0.023	0.005
SVM	0.009	0.021
MLP	0.019	0.015

An important observation is that, although the mean ROC-AUC across the five folds for Random Forest with fingerprints and SVM with MolFormer embeddings was similar, the correlation between their predicted probabilities was 0.7483 (Table 3), indicating partially overlapping but non-identical predictive patterns, suggesting potential complementarity between the models. Pearson correlation between predicted probability scores was chosen as the measure of inter-model agreement rather than a label-based statistic such as Matthews Correlation Coefficient (MCC), because it operates directly on the continuous output of each classifier and is therefore independent of any fixed decision threshold. Given that RF-Fingerprints and SVM-MolFormer produce probability scores with distinct distributions, a threshold-independent measure provides a more reliable characterization of the degree to which the two models encode complementary predictive information.

**Fig. 4:**
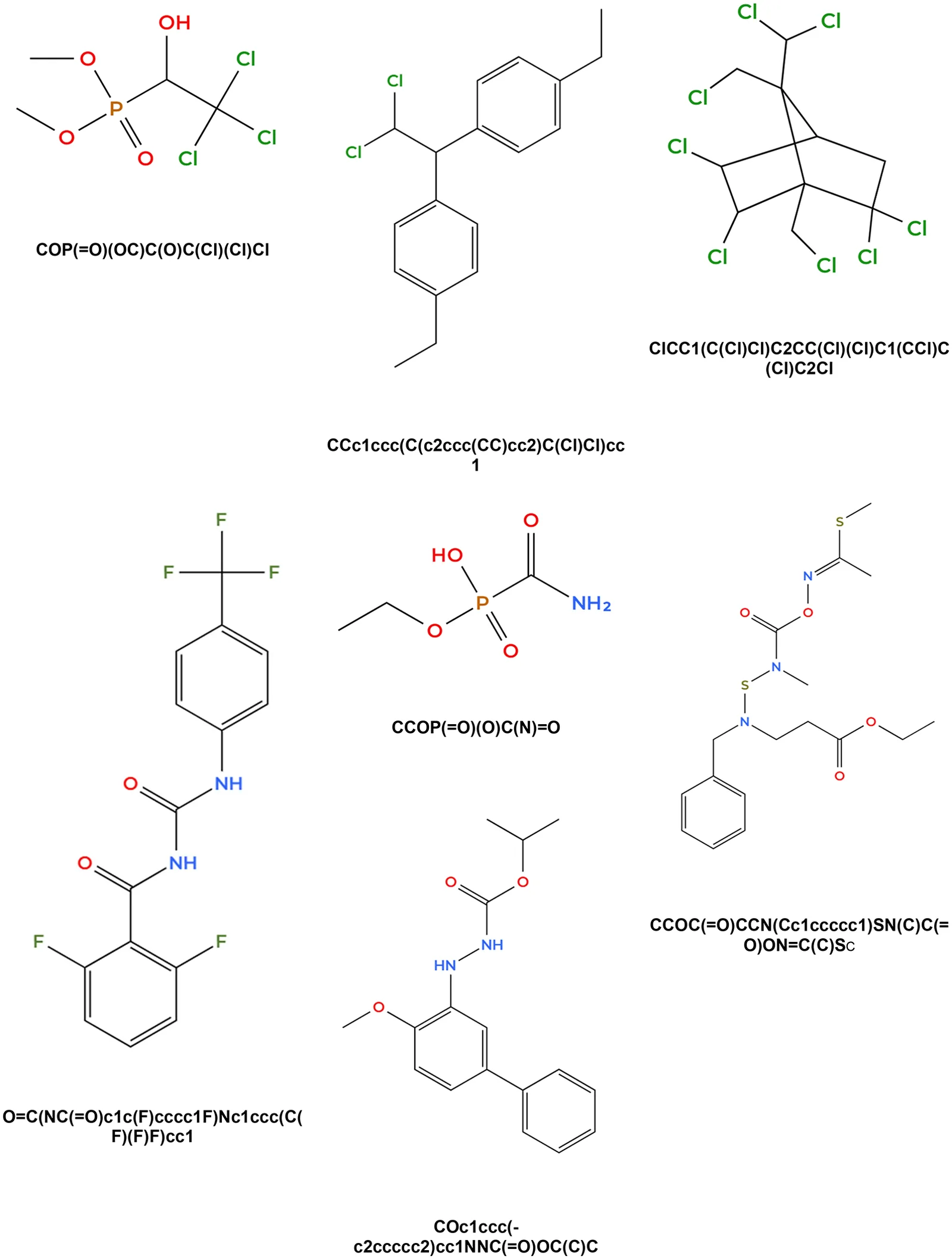
Molecular structures corresponding to SMILES strings correctly classified by SVM-MF

The per-fold AUC-ROC values reported in Table 3 reveal that the lower inter-model agreement observed in Fold 5 (correlation = 0.6417) does not reflect degradation in predictive performance, as both models maintained strong AUC-ROC values in that fold (RF-Fingerprints = 0.862; SVM-MolFormer = 0.840), consistent with the overall cross-validation results. Across all folds, there were instances in which the predictions were correct for the SVM with MolFormer embeddings but incorrect for the Random Forest with fingerprints, and vice versa. This divergence is particularly evident in Fold 5, where reduced correlation coincides with sustained high performance by both models, suggesting that each representation captures distinct and complementary aspects of the chemical space in that partition. To provide qualitative insight, seven representative cases were selected where the embedding-based approach produced correct predictions while the fingerprint-based model failed. Figure 4 shows the molecular structures corresponding to SMILES strings correctly classified by SVM-MF. It is noticeable that the compounds possess phosphate or carbamate groups, or are polyhalogenated with aromatic systems, which correspond to structural motifs frequently associated with pesticide activity. They are consistent with well-established modes of action, such as acetylcholinesterase inhibition and modulation of neuronal ion channels, as well as physicochemical properties linked to persistence and bioaccumulation, which may facilitate recognition by representation-learning models.

The mechanistic basis for SVM-MolFormer’s advantage on these compounds merits further consideration. Although phosphate ester and carbamate functional groups are, in principle, representable within Morgan fingerprints, their circular substructure patterns at radii *r* = 1 and *r* = 2 encode the immediate atomic neighborhood of each motif, the discriminative power of these bits is inherently constrained by the local nature of fingerprint encoding. Specifically, Morgan fingerprints encode substructure presence without capturing the global molecular context in which the functional group is situated: two compounds sharing a phosphate ester moiety but differing substantially in their surrounding scaffold will generate partially overlapping fingerprint vectors, reducing the classifier’s ability to distinguish the contribution of scaffold context to toxicity. Additionally, the binary hashing of Morgan fingerprints is subject to bit collisions, where structurally distinct substructures may map to the same bit position, limiting effective information density particularly for high-radius circular patterns. MolFormer’s bidirectional self-attention mechanism, by contrast, encodes each atomic position in relation to all other tokens in the SMILES sequence simultaneously, producing a holistic representation in which the toxicophoric group is described in the context of the complete molecular architecture. This enables the model to capture the joint contribution of distributed structural features, the spatial and electronic context of the phosphate or carbamate group within the broader scaffold, which collectively determine toxicity in ways that local substructure encoding cannot resolve.

**Fig. 5:**
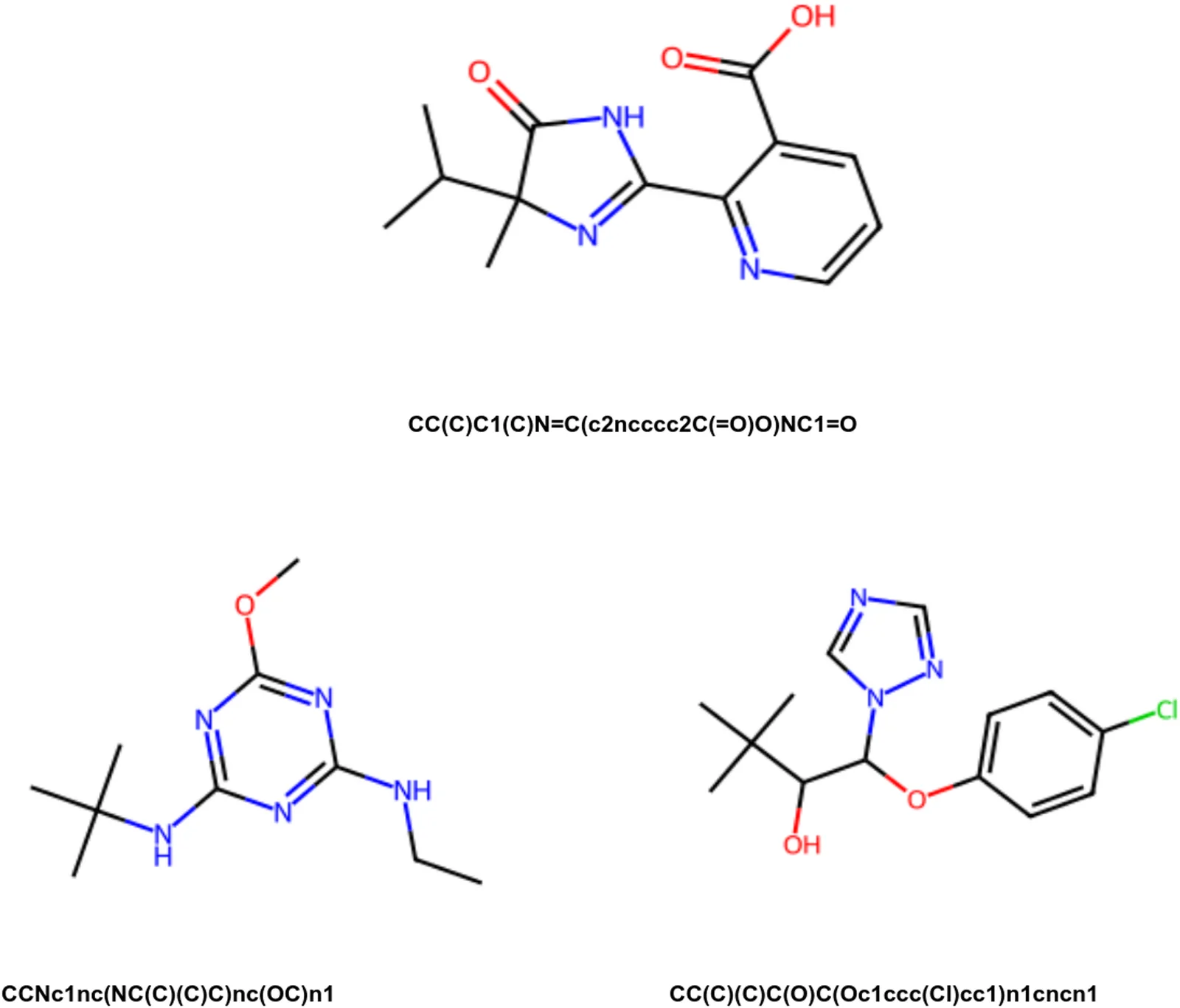
Molecular structures corresponding to SMILES strings correctly classified by RF-Fingerprints but misclassified by SVM-MolFormer

To provide a balanced assessment, representative cases where RF-Fingerprints produced correct predictions while SVM-MolFormer failed were also examined. Figure 5 shows the molecular structures corresponding to SMILES strings correctly classified by RF-Fingerprints but misclassified by SVM-MolFormer. These cases predominantly comprised compounds belonging to well-defined agrochemical classes with established low toxicity to honey bees, including an imidazolinone-derived herbicide, a triazine-based herbicide, and a triazole fungicide. For such compounds, the presence of class-defining substructural motifs (imidazolinone cores, triazine rings, and triazole heterocycles) constitutes a strong and locally encodable signal that Morgan fingerprints capture efficiently through their circular substructure representation. In these cases, the toxicity class is largely determined by the chemical family identity rather than by complex long-range intramolecular interactions, rendering the holistic contextual encoding of MolFormer less critical and the local substructure-based representation of Morgan fingerprints sufficient for correct classification. These findings indicate that classical fingerprint representations retain a competitive advantage in structural regimes where chemical class membership is the dominant predictor of toxicity outcome.

In this sense, we point out that SVM-MF therefore appears to be a strong complementary component within ensemble predictive frameworks, supporting the development of practical platforms for bee toxicity prediction.

**Table 3:** Per-fold AUC-ROC for RF-Fingerprints and SVM-MolFormer, and Pearson correlation between their predicted probability scores across the five folds

Fold	AUC-ROC RF (fingerprints)	AUC-ROCSVM (MolFormer)	Correlation
1	0.873	0.873	0.7963
2	0.913	0.919	0.7872
3	0.829	0.792	0.7953
4	0.850	0.869	0.721
5	0.862	0.840	0.6417
AVG	0.866	0.859	0.7483

Overall, these findings indicate that classical QSAR representations remain strong baselines for honey bee toxicity prediction, with Morgan fingerprints achieving the best performance when paired with Random Forest. Notably, MolFormer embeddings consistently outperformed PaDEL descriptors across all three classifiers (RF, SVM and MLP), reinforcing the advantage of transfer-learned representations over handcrafted descriptors in this setting. Importantly, MolFormer embeddings, used as frozen, pre-trained features from a reduced-scale checkpoint achieved near-parity with the best baseline when combined with SVM, supporting the hypothesis that transfer learning with chemical language models can be an effective and practical strategy for ecotoxicological QSAR under limited labeled data. These results contribute to a broader ongoing research effort in our group toward computational tools for honey bee toxicity prediction, which has also explored graph neural network-based QSAR modeling using the same ApisTox benchmark (Damião et al. 2025)

## Conclusions

This study demonstrated that transfer learning with chemical language models, specifically MolFormer, provides a robust and efficient alternative for predicting acute toxicity in *Apis mellifera*. When compared with classical QSAR representations, MolFormer embeddings not only outperformed PaDEL descriptors across all evaluated classifiers but also achieved near parity with Morgan fingerprints, reaching a ROC-AUC of 0.859 when paired with an SVM.

The main advantage of this approach lies in simplifying the modeling pipeline, eliminating the need to integrate multiple descriptor libraries and to perform complex variable selection procedures. These results support the practical value of pretrained chemical representations for rapid ecotoxicological screening, particularly in scenarios with limited labeled data. Future work should explore full-scale models trained on ~ 1.1 billion molecules and incorporate chronic toxicity endpoints to further improve predictive performance.

The observed complementarity between SVM-MolFormer and RF-Fingerprints, evidenced by partially non-overlapping predictive patterns across folds, naturally motivates the construction of ensemble frameworks as a promising direction for future work. Several combination strategies merit investigation, including majority voting and probability averaging between representation–classifier pairs, stacking approaches using a meta-learner trained on the outputs of individual models, and broader consensus ensembles incorporating graph neural network-based architectures trained on the same benchmark. Beyond performance gains, such ensemble systems could serve as the foundation for unified predictive platforms made available to the scientific and general communities through online tools and mobile applications, contributing to more robust computational screening for pollinator protection.

It should be noted that the present models were developed and evaluated exclusively within the chemical space represented by the ApisTox dataset, which defines their operational applicability domain. Extrapolation to structurally dissimilar chemical classes not represented in this benchmark should be approached with caution. Formal applicability domain analysis, for instance, based on leverage statistics, k-nearest neighbor distance in embedding space, or confidence interval estimation from cross-validation variance, is recommended as a priority step prior to regulatory deployment.

## Data Availability

The data used in this study are publicly available. Honey bee toxicity data were obtained from the ApiSTox dataset deposited in Zenodo (Adamczyk et al., 2024; DOI: 10.5281/zenodo.11062076). Molecular representations used in this study were derived from the SMILES information available in that dataset.
